# A Natural Genetic Variant of Granzyme B Confers Lethality to a Common Viral Infection

**DOI:** 10.1371/journal.ppat.1004526

**Published:** 2014-12-11

**Authors:** Christopher E. Andoniou, Vivien R. Sutton, Matthew E. Wikstrom, Peter Fleming, Kevin Y. T. Thia, Antony Y. Matthews, Dion Kaiserman, Iona S. Schuster, Jerome D. Coudert, Preethi Eldi, Geeta Chaudhri, Gunasegaran Karupiah, Phillip I. Bird, Joseph A. Trapani, Mariapia A. Degli-Esposti

**Affiliations:** 1 Immunology and Virology Program, Centre for Ophthalmology and Visual Science, The University of Western Australia, Crawley, Western Australia, Australia; 2 Centre for Experimental Immunology, Lions Eye Institute, Nedlands, Western Australia, Australia; 3 Cancer Immunology Program, Peter MacCallum Cancer Centre, East Melbourne, Victoria, Australia; 4 Sir Peter MacCallum Department of Oncology, The University of Melbourne, Parkville, Victoria, Australia; 5 Department of Biochemistry and Molecular Biology, School of Biomedical Sciences, Monash University, Clayton, Victoria, Australia; 6 Infection and Immunity Group, Department of Immunology, The John Curtin School of Medical Research, Australian National University, Canberra, Australian Capital Territory, Australia; McMaster University, Canada

## Abstract

Many immune response genes are highly polymorphic, consistent with the selective pressure imposed by pathogens over evolutionary time, and the need to balance infection control with the risk of auto-immunity. Epidemiological and genomic studies have identified many genetic variants that confer susceptibility or resistance to pathogenic micro-organisms. While extensive polymorphism has been reported for the granzyme B (*GzmB*) gene, its relevance to pathogen immunity is unexplored. Here, we describe the biochemical and cytotoxic functions of a common allele of GzmB (GzmB^W^) common in wild mouse. While retaining ‘Asp-ase’ activity, GzmB^W^ has substrate preferences that differ considerably from GzmB^P^, which is common to all inbred strains. *In vitro*, GzmB^W^ preferentially cleaves recombinant Bid, whereas GzmB^P^ activates pro-caspases directly. Recombinant GzmB^W^ and GzmB^P^ induced equivalent apoptosis of uninfected targets cells when delivered with perforin *in vitro*. Nonetheless, mice homozygous for GzmB^W^ were unable to control murine cytomegalovirus (MCMV) infection, and succumbed as a result of excessive liver damage. Although similar numbers of anti-viral CD8 T cells were generated in both mouse strains, GzmB^W^-expressing CD8 T cells isolated from infected mice were unable to kill MCMV-infected targets *in vitro*. Our results suggest that known virally-encoded inhibitors of the intrinsic (mitochondrial) apoptotic pathway account for the increased susceptibility of GzmB^W^ mice to MCMV. We conclude that different natural variants of *GzmB* have a profound impact on the immune response to a common and authentic viral pathogen.

## Author Summary

Granzymes (Gzm) are serine proteases expressed by cytotoxic T cells and natural killer cells, and are important for the destruction of virally infected cells. To date, the function of these molecules has been assessed exclusively in common laboratory mouse strains that express identical granzyme proteins. In wild mouse populations, variants of granzyme B have been identified, but how these function, especially in the context of infections, is unknown. We have generated a novel mouse strain expressing a granzyme B variant found in wild mice (GzmB^W^), and exposed these mice to viral infections. The substrates cleaved by GzmB^W^ were found to differ significantly from those cleaved by the GzmB^P^ protein, which is normally expressed by laboratory mice. Alterations in substrate specificity resulted in GzmB^W^ mice being significantly more susceptible to infection with murine cytomegalovirus, a common mouse pathogen. Our findings demonstrate that polymorphisms in granzyme B can profoundly affect the outcome of infections with some viral pathogens.

## Introduction

Cytotoxic lymphocytes, such as natural killer (NK) cells and CD8 T cells, are essential for the elimination of tumour cells or cells infected with intracellular pathogens. One mechanism cytotoxic lymphocytes utilize to initiate the destruction of target cells is the exocytosis of granules containing perforin (Pfp) and a family of serine proteases known as granzymes (Gzms) [1]. Pfp facilitates the entry of Gzms into the cytoplasm of target cells, where the Gzms cleave specific proteins triggering death of the target. Multiple Gzms have been identified in both humans and the mouse, with GzmA and GzmB being the most abundant and best characterized in both species. While non-cytotoxic functions of Gzms have been described, inducing target cell death appears to be a major function of GzmA and GzmB, and the increased sensitivity of mice lacking these proteins to infection with ectromelia virus (ECTV) and murine cytomegalovirus (MCMV) has been attributed to the role of the Gzms in the killing of infected cells [2]–[4]. Unlike GzmB, which is universally agreed to induce apoptosis [5], the mechanism employed by GzmA to induce cell death remains controversial [6]–[8]; however, it is agreed that this mechanism does not require activated caspases.

Human and mouse GzmB share extensive sequence homology and thus were predicted to kill cells by the same mechanism. However, amino acids that influence substrate binding differ between human and mouse GzmB, with the two proteins now recognized to have different substrate preferences [9]–[11]. A significant difference between the two proteins is that human, but not mouse GzmB, efficiently cleaves the BH3-only protein Bid [10], [12], [13]. Once cleaved, tBid is capable of inducing permeabilization of the mitochondrial outer membrane (MOMP) resulting in the release of pro-apoptotic mediators that ultimately activate a caspase cascade. The finding that cells lacking Bid or overexpressing Bcl-2 survive treatment with human GzmB is consistent with the theory that human GzmB indirectly activates caspases [12], [14], [15]. By contrast, mouse GzmB appears to mediate its effects by directly processing pro-caspases to their active form, and does not require MOMP in order to induce apoptosis [9], [10]. Thus, while both human and mouse GzmB efficiently induce the death of target cells, they achieve this by different mechanisms.

Many pathogens inhibit apoptotic pathways as a means of survival. The differences in mouse and human GzmB substrate specificity may therefore have arisen in response to pathogens targeting different apoptotic pathways in humans and mice. Alternatively, the need to directly target proteins produced by species-specific pathogens could have driven the divergence in GzmB substrate specificities. For example, GzmB inhibits the reactivation of HSV-1 by cleaving the virally encoded ICP4 protein [16]. Similarly, GzmH and GzmB cooperate to suppresses the spread of human adenovirus V by degrading viral proteins essential for replication [17]. Further evidence that selective pressure from pathogens has contributed to changes in GzmB has come from the finding that GzmB polymorphisms exist. In humans, a limited degree of GzmB polymorphism has been described [18], however, the significance of this finding is unclear as there is no difference in the proteolytic activities of the two common alleles and both have equivalent biochemical and cytotoxic functions, at least *in vitro* [19]. In the mouse, 13 common inbred laboratory mouse strains that were tested show no GzmB sequence variation, a finding that probably reflects the limited gene pool from which laboratory strains were derived [20]. By contrast, significant variation in the GzmB sequence was noted in wild mice or wild-derived inbred mouse strains, including in some of the residues that line the substrate cleft. Interestingly, the single allele found in all the inbred mouse strains was relatively uncommon in the wild, found in <20% of isolates [20].

Responses to pathogen challenges have been investigated almost exclusively using inbred mouse strains including knock-out mice rendered genetically deficient in GzmB, but the important question as to whether polymorphisms in GzmB influence the outcome of infections with common pathogens has not been addressed. Here, we have characterized a GzmB allele present in wild mice and found that although its substrate specificity differs from that of GzmB encoded by C57BL/6 (B6) mice, its *in vitro* cytotoxic potential is identical to that of the B6 allele. Nevertheless, substitution of the GzmB allele encoded by B6 mice with the GzmB allele from wild mice led to the inability to effectively control MCMV infection. These data provide novel insights about the relevance of GzmB polymorphisms and demonstrate that polymorphisms in GzmB significantly influence the response to infection with a common, natural viral pathogen.

## Results

### GzmB alleles have different substrate preferences

**Figure 1:**
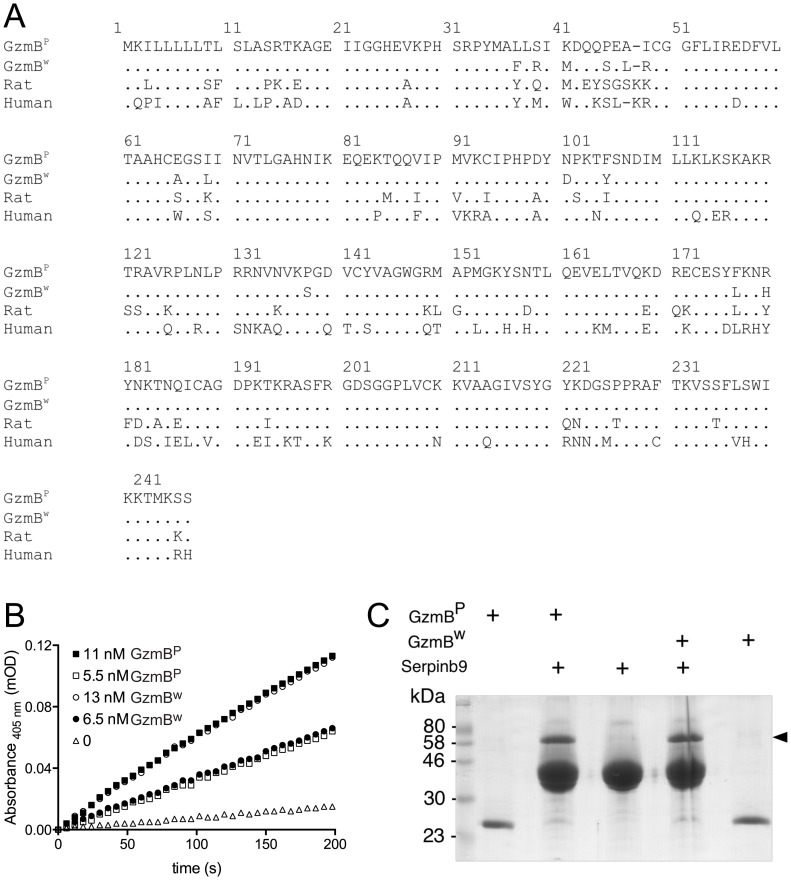
GzmB^W^ and GzmB^P^ have distinct substrate preferences. (**A**) Predicted amino acid sequences of the p (common inbred) and w (wild) alleles of mouse GzmB. A comparison with the sequences of human (RAH allele) and rat GzmB is shown, commencing with the 18-residue leader peptide and the activation peptide GE which is trimmed in the cytotoxic granules by cathepsin C or H. The mature protein commences with the tetrapeptide IIGG. Single amino acid code is used, with dots denoting identical amino acid at the same position. (**B**) Progress curves of recombinant granzymes at indicated concentrations cleaving the peptide thioester substrate Boc-Ala-Ala-Asp (AAD)-SBzl. (**C**) Interaction of recombinant granzymes with Serpinb9. One µg of granzyme was incubated with 10 µg serpin and complex formation assessed by 10% SDS-PAGE with Coomassie Blue staining. Note that all the granzyme shifts into complex (arrowed), indicating fully active preparations.

We had previously shown that the mouse *GzmB* gene is highly polymorphic amongst outbred mouse populations and that some of the polymorphic residues are predicted to impinge on the substrate binding pocket and to potentially influence fine protease specificity [20]. To investigate this further, we selected a wild (w) mouse GzmB allele that is markedly divergent from the allele common to B6 mice as well as the 13 inbred mouse strains we previously typed. For clarity we will refer to the prototype B6 inbred allele as GzmB^P^, and the outbred wild allele as GzmB^W^. GzmB^W^ encodes 13 differences in amino acid sequence from GzmB^P^ or 94.7% amino acid identity over the entire 247 amino acid sequence (**Fig. 1A**).

We expressed and purified recombinant GzmB^W^ and GzmB^P^ in *Pichia pastoris* yeast cells, as previously described [21]. Both forms were indistinguishable in their ability to cleave the generic GzmB substrate AAD-SBzl (**Fig. 1B**), indicating that GzmB^W^ possesses classic Asp-ase activity. Both forms also bound Serpinb9, the intracellular inhibitor of GzmB [22]–[24](**Fig. 1C**), suggesting that GzmB^P^ and GzmB^W^ are subject to similar regulation *in vivo*. Inhibition of any serine proteases by its cognate serpin can only occur if the protease can correctly recognize and cleave the relatively unstructured reactive site loop of the serpin. This further confirmed the proteolytic activity of GzmB^W^, and its cleavage after aspartate [22].

We have previously shown that GzmB^P^ differs from human GzmB in that it cleaves peptide substrates based on Bid (e.g. Abz-IEPDSESQK-dnp) very poorly, and prefers substrates with an aromatic P2 residue and glycine at P2′ ([10] and **Table 1**). Strikingly, GzmB^W^ cleaves peptide substrates based on Bid over 100 times more efficiently than GzmB^P^, and three-fold more efficiently than human GzmB (**Table 1**). Like GzmB^P^, GzmB^W^ prefers substrates with glycine at P2′, but cleaves substrates with an aromatic P2 residue four- to five-fold less efficiently. To confirm these differences in relation to broader substrate specificity, both forms were used in a substrate phage display experiment on a library with Asp fixed at the P1 position [10]. The results indicated very similar requirements at the P4 position (I/L), as well as a requirement for glycine at P2′ (**Figure S1**). However, the strong preference of GzmB^P^ for aromatic residues at P2 was not conserved in GzmB^W^, consistent with the peptide substrate results (**Table 1**).

**Table 1:** GzmB^W^ has a substrate preference similar to human GzmB.

Substrate		*k*_cat_(s^−1^)	*K*_M_ (mM)	*k*_cat_/*K*_M_ (M^−1^s^−1^)
**Human GzmB**				
Abz-IEPD SESQK-dnp	(JNI-7)	1.316±0.091	18.96±2.45	69 397±10 161
Abz-IEPD SGSQK-dnp	(JNI-9)	5.106±0.270	22.06±2.67	231 449±30 585
Abz-LEYD LGALK-dnp	(JNI-13)	0.035±0.002	122.90±6.83	285±20
**Mouse GzmB^P^**				
Abz-IEPD SESQK-dnp	(JNI-7)	0.049±0.004	30.88±2.48	1 575±172
Abz-IEPD SGSQK-dnp	(JNI-9)	0.651±0.182	59.67±2.92	10 915±3101
Abz-LEYD LGALK-dnp	(JNI-13)	2.510±0.160	23.48±2.68	106 948±18 094
**Mouse GzmB^W^**				
Abz-IEPD SESQK-dnp	(JNI-7)	6.14±0.54	30.32±5.60	202 364±17 877
Abz-IEPD SGSQK-dnp	(JNI-9)	24.45±1.31	21.22±2.35	1 152 328±61 606
Abz-LEYD LGALK-dnp	(JNI-13)	1.97±0.07	83.80±5.06	23 573±1 649

**Figure 2:**
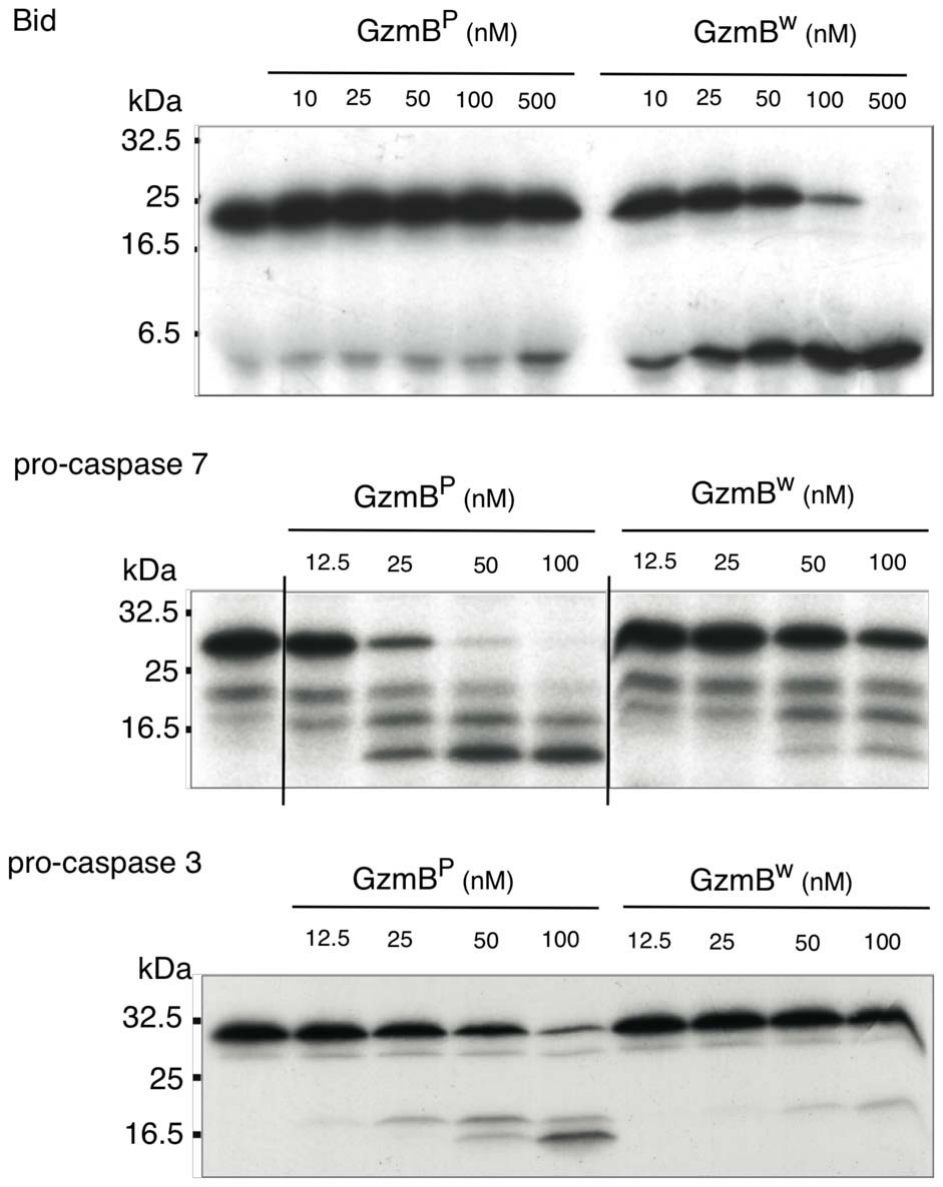
GzmB^W^ cleaves Bid more efficiently and procaspases less efficiently than GzmB^P^. *In vitro* translated ^35^S-labeled mouse procaspase 3, mouse procaspase 7, or mouse Bid were incubated with the indicated amounts of granzymes at 37°C for 30 min. Products were separated by 15% SDS-PAGE and visualized by fluorography.

**Figure 3:**
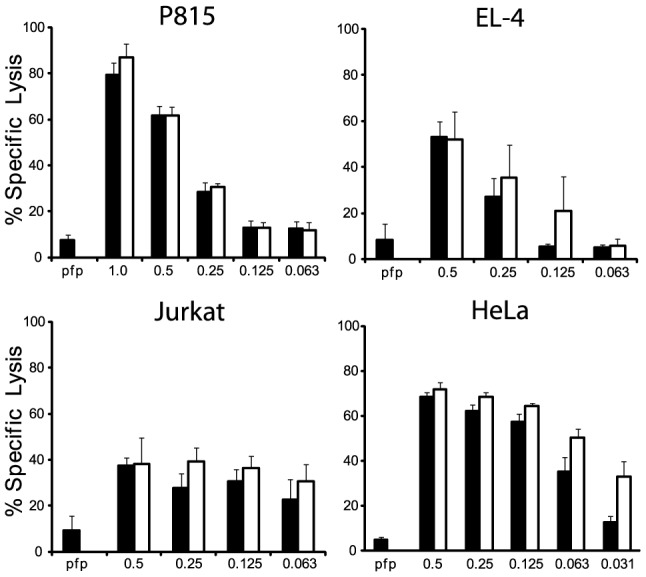
GzmB^W^ and GzmB^P^ have equivalent cytotoxic potential *in vitro*. (**A**) Mouse P815, (**B**) mouse EL-4, (**C**) human Jurkat and (**D**) HeLa cells were labeled with ^51^Cr, and exposed to sub-lytic concentrations of recombinant perforin in combination with either mouse p (black bars) or w (white bars) recombinant GzmB. Specific ^51^Cr release is shown as the mean of individual experiments (each performed in triplicate) ± SEM. The number of experiments performed were: P815 (n = 5), EL-4 (n = 2), Jurkat (n = 3) and HeLa (n = 4).

We next examined whether the differences in turnover of Bid (IEPD) substrates is also reflected in a different capacity to cleave intact Bid or effector pro-caspases 3 and 7. We found that GzmB^W^ is 50–100 fold more efficient than GzmB^P^ at cleaving recombinant Bid, but far less efficient at activating pro-caspase-3 or pro-caspapse-7 (**Fig. 2**). To determine whether these changes in substrate preference translate into a variable capacity to kill target cells, we exposed P815 (mouse mastocytoma), EL-4 (mouse thymoma), HeLa (human cervical cancer) and Jurkat (human T lymphoma) cells to graded doses of each GzmB form and very low (‘sublytic’) quantities of purified recombinant Pfp (**Fig. 3**). For each cell line, we found no significant difference in susceptibility to apoptosis. Overall, we concluded that there is no significant difference in the intrinsic pro-apoptotic activity of GzmB expressed by the inbred laboratory mice and outbred wild mice.

### GzmB^W^ from T and NK cells retains full functionality

To examine the role of the GzmB^W^ allele in more physiological settings, we generated a congenic mouse strain carrying the w allele. This was achieved by backcrossing the w/w mouse for >20 generations with B6, at each generation selecting for the w allele. This strain was also crossed with B6.OT1 mice to create the GzmB^W/W^.OT1 strain, so that antigen restricted CTLs could be studied in both strains (see below). An alternative and probably less laborious approach to deriving the congenic line over so many generations would have been to develop a ‘knock in’ of the W allele on P strain. However, we and other groups have not been successful in this approach, due to the large number of highly homologous granzyme gene sequences closely linked in the *GzmB* locus.

Using whole exome DNA sequencing we confirmed that the *GzmB* locus on chromosome 14 including the *GzmB* gene, all 6 linked *Gzm* genes and the gene encoding Cathepsin G (expressed in neutrophils, but not cytotoxic lymphocytes) were derived from w. The genetic interval derived from w comprised approximately 18% of the chromosome 14 DNA. Overall, the genetic content of the w/w mouse was >99.1% derived from B6. Having derived the DNA sequence of the 0.9% of the genome that remained from the w strain, we were able to assess whether the high degree of polymorphism identified for *GzmB* was also the case for the other granzymes (*GzmC-G and N*). This was not the case: whereas >5.3% of the amino acids of GzmB differed between the two allotypes, the corresponding figure across the other 6 genes was <0.3% (a total of four polymorphic residues out of 1362 across the 6 coding regions; P<0.0001) (**Table 2**).

**Table 2:** Non-synonymous polymorphisms in *Gzm* genes linked to *GzmB on Chr 14* 1.

Gene	Polymorphism^3^
*GzmC*	L7I^4^
	**E175K (rs266005675)** 2
*GzmF*	I4V (rs49622871)^2^^,^ 4
*GzmN*	None
*GzmG*	**G51R**
	**S144T (rs244881887)** 2
*GzmD*	I4V^4^
	**W247R (rs16803648)** 2
*GzmE*	None

1 Genes are listed in their correct order on Chr 14, not alphabetically.

2 Previously recorded in various SNP databases.

3 Most of the predicted amino acid changes are highly conservative; unlike GzmB, none are predicted to affect the substrate cleft.

4 Map to the leader sequence, so do not affect the mature protein; a total of four polymorphisms affecting mature protein are shown in bold.

**Figure 4:**
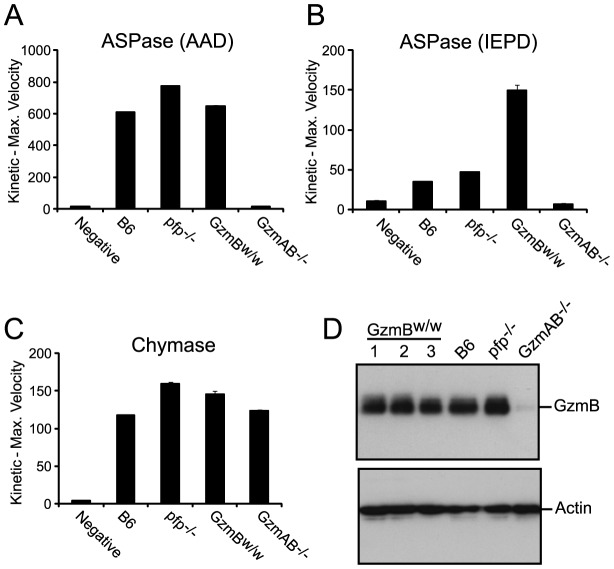
Granule enzyme activity and GzmB expression in T cell lysates from GzmB^W/W^ and GzmB^P/P^ mice. (**A**) GzmB (ASPase) activity was measured as the maximum rate of cleavage of the peptide thioester substrate Boc-Ala-Ala-Asp (AAD)-SBzl or (**B**) the activity detected through cleavage of Ac-IEPD-pNA. (**C**) Chymotrypsin-like (chymase) activity (cleavage of Suc-Phe-Leu-Phe SBzl) was used as an independent measure of granule enzyme activity. The data points show the mean ± SEM of triplicate readings. The data is representative of 3 individual experiments. Equivalent results were obtained with lysates generated from IL-2 activated NK cells. (**D**) Western blot analysis for GzmB expression in OT1 T cells from outbred w/w mice, B6 and control GKO (B6.Pfp^−/−^ and B6.GzmAB^−/−^) mice. The blot was re-probed for actin expression.

We wished to establish beyond doubt that our *in vitro* enzymatic findings, which were derived with purified recombinant proteases, would be replicated in *bona fide* antigen restricted CTLs, which, along with NK cells, are the authentic physiological context for granzyme expression. We therefore generated OVA^257^ specific activated T cells from the spleens of B6 and GzmB^W/W^ congenic OT1 mice and tested the resultant cell lysates on peptide substrates. The generic Asp-ase substrate AAD-SBzl was cleaved with similar efficiency by B6 and w/w T cell lysates (**Fig. 4A**). By contrast, the tetrapeptide substrate IEPD was cleaved more efficiently by w/w lysate (with >3 times the maximum velocity of p/p, p<0.05), confirming differences in the fine specificity of the two alloforms of GzmB observed with *in vitro*-generated proteins (**Fig. 4B**). Structural predictions for the granzymes whose genes are closely linked to *GzmB*, indicate that these proteases should have chymotrypsin-like (‘chymase’) activity and cleave after hydrophobic P1 residues [25] as has been clearly demonstrated for the human orthologue GzmH [26]. As expected, the respective CTL lysates from the w and p mice had indistinguishable chymase activity (**Figure 4C**), further confirming that the minimal changes in amino acid sequence among the chymase granzymes had no impact on substrate preference. There was no turnover in AAD or IEPD in T cells derived from GzmAB-null mice, while B6 mice deficient in perforin (whose gene maps to Chr 10) showed similar activity to wild type B6 mice (**Fig. 4A and C**). Western blot of cell lysates also showed no quantitative difference in GzmB expression across the various strains tested (**Fig. 4D**).

### GzmB^W/W^ mice show increased sensitivity to MCMV infection

**Figure 5:**
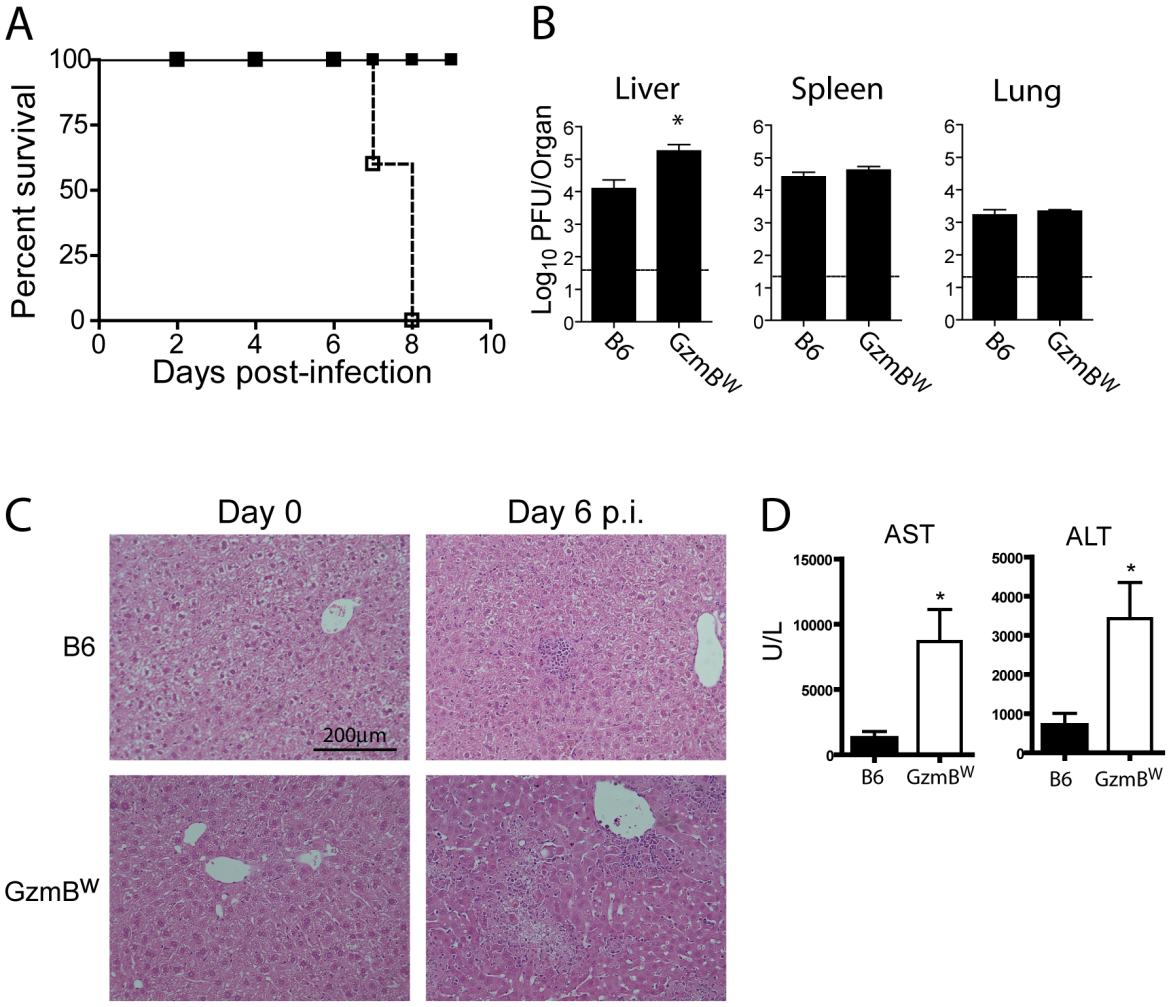
GzmB^W/W^ mice are sensitive to infection with Δm157 MCMV. (**A**) B6 mice (black square) or GzmB^W/W^ mice (white square) were infected with 2×10^4^ pfu of MCMV Δm157 and survival monitored over the indicated time course (n = 5 for each group). ***P<0.0001. (**B**) The indicated mouse strains were infected with 2×10^4^ pfu of MCMV Δm157, the indicated organs were removed at day 7 post-infection, and viral load quantified by plaque assay. Data are pooled from 2 independent experiments, mean ± SEM are plotted, where n≥8. *P<0.05. (**C**) Livers from uninfected or MCMV-infected mice were isolated at day 6 post-infection, fixed and tissue sections stained with haematoxylin and eosin. The results are representative of two independent experiments. (**D**) Liver enzymes in the serum of B6 mice (black bar) or GzmB^W/W^ mice (white bar) were measured at day 6 post-infection. Data are pooled from two independent experiments where n≥5. *P<0.05.

Our *in vitro* analyses determined that the substrate specificity of GzmB^W^ differs from that of the B6 allele. Since the Gzms have been shown to play a critical role in viral infections, we next examined whether the differences in substrate specificity observed *in vitro* and *ex vivo* can lead to functional differences after infection with bona fide mouse pathogens. MCMV, a natural pathogen of mice, is partly controlled by activities mediated by GzmB [4]. Thus, the effect of GzmB^W^ on the ability of the host to control MCMV infection was investigated. In B6 mice, NK cells rapidly control MCMV infection via activation mediated by engagement of the Ly49H activating NK cell receptor. However, in the wild most (≥80%) MCMV variants encode m157 proteins that are unable to activate NK cells [27] and indeed, the frequency of Ly49H-resistance is rare in outbred wild mice [28]. These findings indicate that B6-like Ly49H-m157 interactions are not a feature of host–MCMV interactions in the wild. Thus, to examine the role of GzmB^W/W^ in a setting that reflects the situation in wild mouse populations, we utilized a virus lacking the m157 viral protein (Δm157). In the absence of m157, MCMV replicates to high titers in the visceral organs of B6 mice, and is eventually controlled primarily by cytotoxic CD8 T cells. Unlike B6 mice (GzmB^P/P^), infection of the GzmB^W/W^ mice with Δm157 MCMV resulted in rapid mortality (**Fig. 5A**). At day 7 post-infection, viral loads in the spleens and lungs of GzmB^W/W^ mice were not significantly different from those observed in B6 mice (**Fig. 5B**). By contrast, the livers of GzmB^W/W^ mice contained approximately 10 fold more virus than livers of B6 mice (**Fig. 5B**). Histological analysis of GzmB^W/W^ livers harvested at day 6 post-infection revealed significant areas of focal necrosis and diffuse cellular infiltrates, while in B6 mice no significant damage was evident in liver sections (**Fig. 5C**). The advanced liver damage observed in GzmB^W/W^ mice by histological analysis was also confirmed by measuring circulating liver transaminase levels. Serum levels of the liver enzymes alanine aminotransferase (ALT) and aspartate aminotransferase (AST) in GzmB^W/W^ mice were significantly elevated at day 6 post-infection (**Fig. 5D**). These data indicate that GzmB^W/W^ mice have an impaired response to Δm157 MCMV infection that manifests as significantly higher viral loads within the liver, and tissue damage to this organ, which markedly increases mortality.

GzmB, along with GzmA, produced by cytotoxic CD8 T cells is essential for host defense against the poxvirus ECTV [2], [29]. ECTV is a large DNA virus that is the causative agent of mousepox. GzmB^W/W^ mice infected with 10^5^ pfu of the Moscow strain of ETCV succumbed to infection at rate equivalent to that of B6 mice (**Figure S2A**), and viral load in the blood of GzmB^W/W^ mice at day 8 post-infection was not significantly different to that of B6 mice (**Figure S2B**). Since mice that lack GzmB are 100-fold more susceptible to ECTV infection [2], these data indicate that GzmB^W^ can substitute for the B6 allele of GzmB in the context of ECTV infection, and provide independent evidence that the GzmB^W^ allele is functional not only *in vitro* (**Table 1**), but also *in vivo*.

### The response of GzmB^W/W^ mice to MCMV infection mirrors that of GzmB-deficient mice

**Figure 6:**
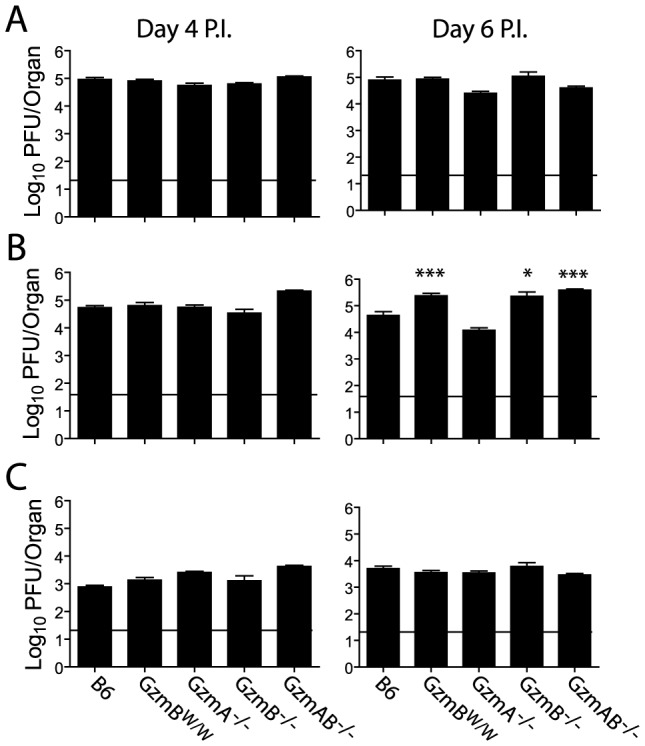
Increased viral load in mice lacking GzmB. The indicated mouse strains were infected with 2×10^4^ pfu of MCMV Δm157. At days 4 and 6 post-infection viral loads in the (**A**) spleen, (**B**) liver and (**C**) lungs were quantified by plaque assay. Data are pooled from 3 independent experiments, mean ± SEM are plotted, where n≥6. *P<0.05.**P<0.005.

The outcome of infection with the Δm157 MCMV virus was then compared in GzmB^W/W^ mice and mice lacking GzmA, GzmB or both GzmA and B. Viral loads were measured in target organs (spleen, liver and lungs) at days 4 and 6 post-infection by plaque assay. Experiments were terminated at day 6 post-infection as GzmB^W/W^ mice become highly sensitive to infection after this time. Viral loads in mice deficient for GzmA were equivalent to those of B6 mice in all organs tested, at both day 4 and 6 (**Fig. 6**), suggesting that, at least during the acute phase of infection, GzmA is not required for viral control. By contrast, viral loads in the livers of mice deficient in GzmB, either alone, or in combination with GzmA, were significantly higher than those observed in B6 mice at day 6 post-infection (**Fig. 6B**). Thus, GzmB is essential for effective control of MCMV Δm157 in the liver. Furthermore, replication of the Δm157 virus in the livers of GzmB^W/W^ mice was equivalent to that of GzmB^−/−^ and GzmA/B^−/−^ mice, indicating that GzmB^W/W^ cannot substitute for the B6 allele during the anti-viral response to MCMV.

### Deregulated cytokine production does not contribute to liver damage in MCMV infected GzmB^W/W^ mice

**Figure 7:**
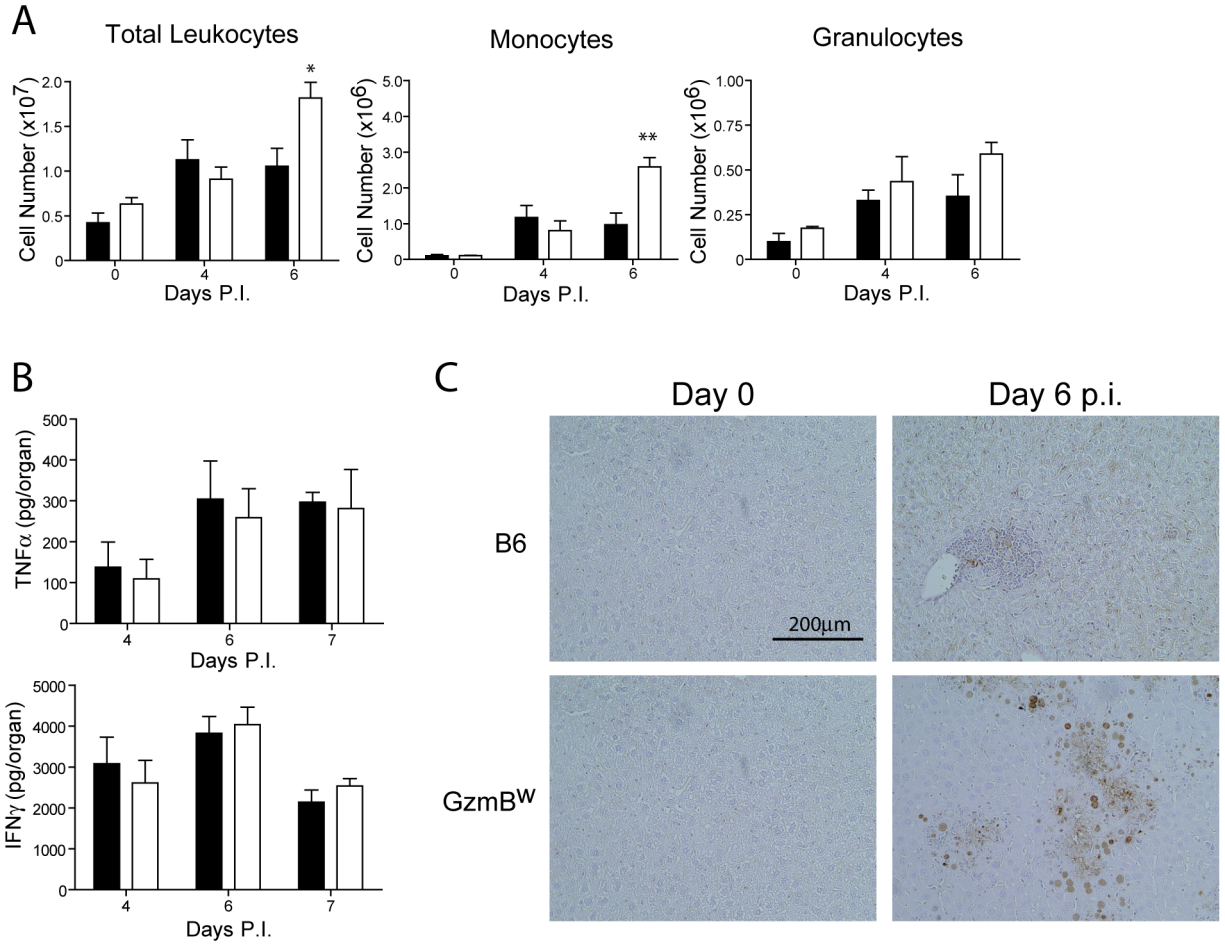
Liver damage in GzmB^W/W^ mice is not the result of immune-mediated pathology. (**A**) B6 mice (black bar) or GzmB^W/W^ mice (white bar) were infected with Δm157 MCMV and total liver leukocytes, inflammatory monocytes, and granulocytes enumerated at the indicated times. Data are pooled from 2 independent experiments, mean ± SEM are plotted, where n≥5. *P<0.05. **P<0.01. (**B**) Levels of TNF-α and IFN-γ in the liver at the indicated times post-infection were quantified by ELISA. (**C**) Livers from uninfected or MCMV-infected mice at day 6 post-infection were stained with the Chroma 101 anti-IE1 antibody followed by detection with a DAB substrate. Sections were counterstained with haematoxylin. The results are representative of two independent experiments.

The effect of the Δm157 virus on GzmB^W/W^ mice is reminiscent of the effects of MCMV infection on pfp-deficient mice. Mice lacking pfp exhibit increased mortality after MCMV infection [30]. While MCMV replication in the liver of pfp^−/−^ mice is significantly higher than that observed in B6 mice, uncontrolled viral replication is not the cause of mortality [4]. Rather, a fatal hemophagocytic lymphohistiocytsis (HLH)-like syndrome develops in pfp^−/−^ mice due to the uncontrolled production of TNFα by accumulating activated macrophages [4]. To determine if GzmB^W/W^ mice exhibited any signs of an HLH-like syndrome, we examined lymphocyte populations and cytokine production after MCMV infection. Following infection with the Δm157 MCMV mutant, the livers of GzmB^W/W^ mice contained significantly more leukocytes at day 6 post-infection (**Fig. 7A**). Analysis of these leukocytes by FACS revealed that the number of inflammatory monocytes (CD11b^+^ Ly6C^+^ Ly6G^−^) in the liver of GzmB^W/W^ mice were significantly increased (**Fig. 7A**). While a similar trend was observed for granulocytes (CD11b^+^ Ly6G^+^), this did not reach statistical significance (**Fig. 7A**). Given the increased numbers of inflammatory monocytes in the livers of GzmB^W/W^ mice, we measured pro-inflammatory cytokine production to determine if this may be contributing to the observed mortality. The levels of TNFα and IFNγ in the livers of GzmB^W/W^ mice were not significantly different from those observed in B6 mice (**Fig. 7B**). These findings indicate that GzmB^W/W^ mice do not develop an HLH-like syndrome following MCMV infection.

In order to better characterize the effects of MCMV infection in GzmB^W/W^ mice, immunohistochemistry (IHC) staining of liver sections was performed. IHC staining of liver sections with an antibody specific for the IE1 protein of MCMV revealed a stark difference between B6 and the GzmB^W/W^ mice. In B6 mice inflammatory foci were evident at day 6 post-infection, consisting of small numbers of infected hepatocytes (brown stain), typically surrounded by a large number of lymphocytes (**Fig. 7C**). In GzmB^W/W^ mice, the inflammatory foci were larger in size and contained significantly more infected hepatocytes (**Fig. 7C**). Furthermore, areas of necrosis and cell debris within the centre of the foci were apparent (**Fig. 7C**). Together the data indicate that the liver damage observed in GzmB^W/W^ mice was the direct result of uncontrolled viral replication, rather than the outcome of immune-mediated pathology.

### CD8 T cells from GzmB^W/W^ mice are unable to kill virally infected cells

The inability of GzmB^W/W^ mice to control MCMV in the liver suggests that these mice may not be generating an appropriate anti-viral CTL response. Serpinb9 is a potent inhibitor of GzmB that is expressed by CTL [24]. Expression of Serpinb9 is required to prevent the premature apoptosis of CTL generated in response to lymphocytic choriomenigitis virus (LCMV) or Listeria monoctogenes infection [31]. We found that the GzmB^W^ protein effectively bound Serpinb9 in an *in vitro* assay (**Fig. 1C**), but this finding does not preclude the possibility that Serpinb9 is unable to efficiently inhibit GzmB^W^ in CTL *in vivo*. We therefore infected *Serpinb9^−/−^* mice with Δm157 MCMV and quantified viral replication. Viral titers in the spleen, liver, and lungs of *Serpinb9^−/−^* mice were not significantly different from those of B6 mice (**Figure S3**). Thus, the effects observed in GzmB^W/W^ mice are not the result of an inability of Serpinb9 to inhibit GzmB^W^.

**Figure 8:**
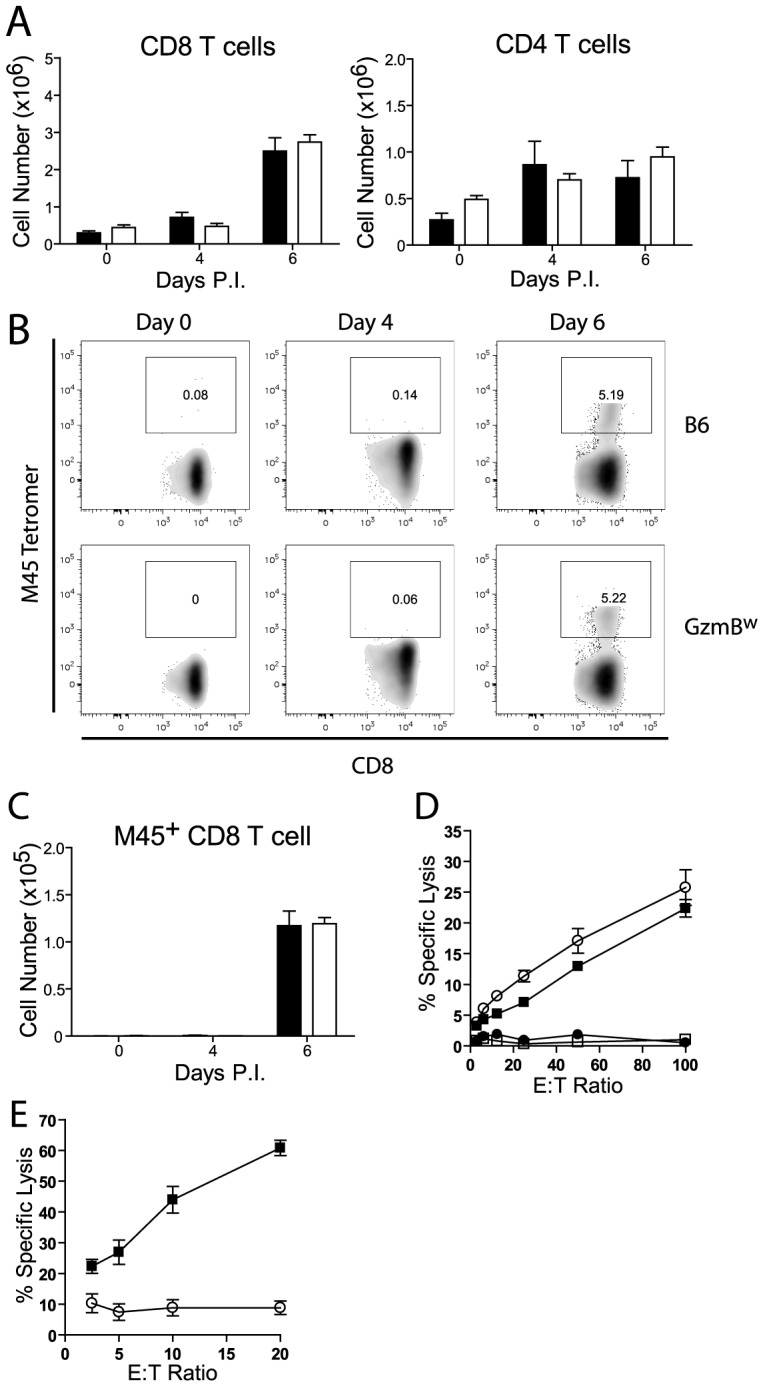
GzmB^W/W^ CD8 T cells are unable to lyse MCMV infected cells. (**A**) The numbers of CD8 and CD4 T cells localizing to the liver of B6 mice (black bar) or GzmB^w/w^ mice (white bar) after infection with MCMV Δm157 are shown. (**B**) At the indicated times post-infection, splenocytes were stained with anti-CD8, anti-TCRβ, and M45 tetramers. Representative FACS plots showing the percentage of M45-specific CD8 T cells are shown, and (**C**) the total numbers of M45-specific CD8 T cells are plotted. Data are pooled from 2 independent experiments, where n≥5. (**D**) Splenocytes were isolated from MCMV Δm157 infected B6 (black square) and GzmB^w/w^ mice (white circle), or from uninfected B6 mice (white square) and GzmB^w/w^ mice (black circle). Splenocytes were cultured with ^51^Cr-labeled M45 pulsed EL4 cells for 4 h and specific lysis determined. n = 6 for each data point. (**E**) CD8 T cells were purified from B6 mice (black square) and GzmB^w/w^ mice (open circle) and co-cultured with MCMV infected IC-21 macrophages for 18 h at the indicated E∶T ratios. n = 5 for each data point.

Next, we assessed the generation and effectiveness of anti-viral T cell responses in GzmB^W/W^ mice. The total numbers of CD8 and CD4 T cells localizing to the livers of GzmB^W/W^ mice after MCMV infection were not significantly different from those observed in MCMV-infected B6 mice (**Fig. 8A**). A peptide derived from the M45 protein of MCMV is the immunodominant epitope recognized by CD8 T cells in B6 mice [32]. The percentage of CD8 T cells stained by an M45 tetramer in B6 mice following MCMV infection was similar to that observed in GzmB^W/W^ mice (**Fig. 8B**), and there were no differences in the total number of M45-specific CD8 T cells generated (**Fig. 8C**). The capacity of M45-specific T cells to kill target cells was also assessed. Splenocytes isolated from B6 mice at day 6 post-infection efficiently lysed M45 pulsed target cells, while no significant lysis was apparent when splenocytes from uninfected mice were used (**Fig. 8D**). The capacity of GzmB^W/W^ splenocytes to lyse M45 pulsed target cells was similar to that of B6 cells (**Fig. 8D**). Hence, GzmB^W/W^ mice generate an effective CD8 T cell response following infection with Δm157 MCMV and these T cells are able to efficiently kill model target cells. In addition to peptide pulsed targets, we tested the capacity of GzmB^W/W^ and GzmB^P/P^ CD8 T cells to kill MCMV-infected cells. CD8 T cells were purified from the spleen of B6 mice or GzmB^W/W^ mice 6 days after infection, co-cultured with MCMV-infected IC-21 macrophages and macrophage viability assessed 18 h later. GzmB^P/P^ CD8 T efficiently lysed the MCMV-infected target cells in a dose dependent manner, whereas GzmB^W/W^ CD8 T cells were almost completely ineffective (**Fig. 8E**). Collectively, the data suggested that CD8 T cells expressing the w allele of GzmB are elicited and activated in response to MCMV infection, but are unable to kill MCMV-infected targets, accounting of the susceptibility of these mice to the virus.

## Discussion

Murine GzmB is highly polymorphic in wild mice, but the physiological relevance of this finding has been unclear. Here, we have characterized the biochemical and physiological function of an allelic variant of GzmB, GzmB^W^, found in wild mice. We have found that GzmB polymorphism affects the substrates cleaved by the protease *in vitro*. GzmB^W^ efficiently cleaved peptide substrates based on the Bid sequence, and Bid itself, and as such, has an activity that is distinct from the GzmB^P^ allele expressed by inbred mouse strains. Despite having different substrate preferences, GzmB^P^ and GzmB^W^ induced apoptosis of uninfected target cells with similar efficiency *in vitro*, and GzmB^W/W^ mice were as efficient as wild type B6 mice in controlling ECTV infection. These data clearly show that the GzmB^W^ isoform is bioactive *in vitro* and, more importantly, *in vivo*, at least as far as the requirements for its critical role in inducing the death of “generic” targets cells and in controlling infection with ECTV.

A striking finding of the study is that B6 mice expressing GzmB^W^ are extremely sensitive to MCMV infection demonstrating that polymorphism in GzmB can have a significant impact on the capacity of the host to control specific pathogens. We found that GzmB was essential for control of Δμ157 MCMV (the most common MCMV variant found in the wild), and that the GzmB^W^ allele was unable to substitute for GzmB^P^ in this setting. These results argue for a defect in cytotoxicity, however, several non-cytotoxic roles have been ascribed to various Gzms. For example, GzmA and GzmM have a role in the production/release of pro-inflammatory cytokines [33]–[35]. We found that production of the pro-inflammatory cytokines IFNγ and TNFα by GzmB^W/W^ mice following MCMV infection was equivalent to that of B6 mice. Furthermore, GzmB^W/W^ mice generated CD8 T cell effectors at the expected frequency with localisation of these cells to the liver equivalent to that observed in B6 mice. Thus, the inability of GzmB^W/W^ mice to control MCMV infection was not the result of defects in the production of pro-inflammatory cytokines, nor was it due to defective CD8 T cell numbers.

The initiation of apoptosis by activating caspases is the best-characterized function of GzmB. Human GzmB initiates apoptosis by activating caspases via two distinct pathways [36]. A mitochondrial-dependent pathway is activated when human GzmB cleaves Bid resulting in MOMP and the release of pro-apoptotic mediators [13], [37]. Mitochondrial-independent pathways operate via direct pro-caspase activation when sufficiently high concentrations of GzmB are delivered to the target cell cytosol [36]. Given the substrate specificity of the w allele, apoptosis induced by this form of GzmB is likely to mirror that of human GzmB. *In vitro*, GzmB^W^ was as effective as GzmB^P^ at inducing apoptosis in uninfected target cells, and anti-viral specific CD8 T cells isolated from GzmB^W/W^ mice killed peptide pulsed target cells efficiently, indicating that there is no intrinsic defect in the direct cytotoxic capacity of GzmB^W^. However, CD8 T cells isolated from GzmB^W/W^ mice were unable to kill MCMV-infected target cells *in vitro*. MCMV encodes potent inhibitors of both Bax and Bak that together prevent MOMP in response to multiple stimuli [38]–[41]. Collectively, the data strongly suggest that as GzmB^W^ preferentially cleaves Bid, rather than directly activating caspases, it is susceptible to inhibition by MCMV-encoded proteins that block the intrinsic cell death pathway.

Our previous work has studied some of the key residues that dictate the species-specific substrate preferences of human and mouse GzmB. We found that two residues whose side-chains impinge on the substrate cleft, ^180^Arg and ^222^Lys are important in this regard, as substitution of the corresponding human or rat residue conferred a greater capacity to cleave Bid [10]. Residue 222 was invariant in all of the mouse GzmB alleles we previously sequenced and is thus common to both the mouse w and p alleles. However at position 180, the w allele encodes His, rather than Arg (present in the p allele), or Tyr which is found in human GzmB. His is also present at the same position in M. casteneus and M. spretus, mouse subspecies commonly found in certain parts of Asia. Given that ^222^Lys is invariant, it is extremely likely that the altered substrate preferences that result in the w allele having a far greater ability to cleave mouse Bid than to activate pro-caspases directly must rely on ^180^His. We found that in contrast to GzmB^P^, we found that GzmB^W^ is unable to effectively activate some pro-caspases directly. Thus, apoptosis induced by GzmB^W^ is reliant on activating the intrinsic pathway. Overall, a combination of changes in GzmB's fine substrate specificity, together with the expression of MCMV-encoded inhibitors of MOMP accounts for the failure of GzmB^W^ expressing CD8 T cells to kill virally infected cells, resulting in uncontrolled viral replication.

In summary, we have demonstrated that a GzmB polymorphism commonly found in the wild has a profound influence on the ability of mice to control a natural pathogen. Importantly, the devastating effects elicited in hosts carrying the GzmB^W^ allele by what is a common viral infection suggest that this allele has been maintained in the population because it confers a survival advantage in a setting yet to be defined. Furthermore, the function of GzmB in the response to pathogens and tumours has been investigated almost exclusively using inbred mouse strains all of which express the same allele of GzmB. The results of this study suggest that the use of mouse strains expressing alternative alleles of GzmB will be important for gaining a full understating of the role played by GzmB during an immune response. These findings also raise the possibility that alleles of GzmB identified in humans could impact on the control of some human pathogens.

## Materials and Methods

### Ethics statement

This study was performed in accordance with the recommendations in the Australian code of practice for the care and use of animals for scientific purposes and the Australian National Health and Medical Research Council Guidelines and Policies on Animal Ethics. Experiments were approved by the Animal Ethics and Experimentation Committee of the University of Western Australia (Protocol # RA3/100/1094), the Animal Ethics and Experimentation Committee of the Peter MacCallum Cancer Centre (Protocol #E381) and the Animal Ethics and Experimentation Committee of the Australian National University (Protocol # A2012/041).

### Protein production

Recombinant granzymes were produced as artificial zymogens in *Pichia pastoris*, activated using enterokinase following purification, and assessed for the ability to cleave the synthetic peptide thiobenzylester (Boc-Ala-Ala-Asp (AAD)-SBzl [10], [21]. A GzmB^w^ cDNA with optimized mouse codon usage was synthesized *in vitro* (GenScript). Specific activity of purified mouse granzymes was assessed by SDS-stable binding to an enhanced form of Serpinb9 (Cys339Asp) produced as described [42]. Preparations were routinely >95% active.

### Production of granzyme substrates *in vitro*

^35^S-labeled mouse procaspase 3 or mouse Bid was produced from cDNAs in the expression vector pSVTf via in vitro transcription and translation [19].

### Phage display

Recombinant granzymes were used to probe a P1 Asp-anchored library, as described [43].

### Generation of wild GzmB mouse lines

A wild mouse colony (B1–6) maintained at the Animal Resource Centre (Canning Vale, WA), which expressed the outbred GzmB allele (GzmB^w/w^) [20] was crossed with C57BL/6 mice. Mice homozygous for GzmB^w/w^ were backcrossed to C57BL/6 for 22 generations. These F22 mice were also crossed with B6.OT1 (ovalbumin-specific, H-2^b^ -restricted T-cell receptor transgenic) mice to generate a GzmB^w/w^.OT1 line.

### Mice

Inbred C57BL/6J (B6) mice were obtained from the Animal Resources Centre (Perth, Western Australia, Australia), orWalter and Eliza Hall Institute (Melbourne). B6.granzymeA^−/−^ (GzmA^−/−^), B6.granzymeB^−/−^ (GzmB^−/−^), B6.granzymeAB^−/−^ (GzmAB^−/−^), B6.perforin^−/−^ (Pfp^−/−^) B6.OT1, B6.Gzm.AB^−/−.^OT1, and B6.pfp.OT1 mice were bred and maintained at the Peter MacCallum Cancer Centre, Melbourne. B6 mice carrying the *Serpinb9^tm1.1/Pib^* allele (Serpinb9^−/−^ mice) were generated and maintained at Monash University [44]. Mice were used at 6–10 weeks of age.

### Generation of primary mouse CTL

OVA^257^ specific activated T cells were generated from the spleens of the various OT1 mice (B6, B6.Pfp^−/−^, B6.GzmAB^−/−^, B6.GzmB^w/w^) as previously described [45]. The cytotoxic activity of the CTL was verified in ^51^Cr release assays using H-2^b^-peptide pulsed target cells (EL-4), as previously described [46].

### Granule enzyme activity assays

Whole cell lysates were prepared from CTL cultures and normalized for protein content. Granule enzyme activity was determined as previously described [47] using synthetic peptide thiobenzylester (Boc-Ala-Ala-Asp (AAD)-SBzl and Suc-Phe-Leu-Phe SBzl) and paranitroanilide (Acetyl-Ile-Glu-Pro-Asp-paranitroanilide, Ac-IEPD-pNA) substrates (SM Biochemicals, CA, USA).

### Western blot

Ten µg of whole cell lysate was separated on NuPAGE 4–12% Bis Tris gels (Life Technologies, CA, USA), transferred and probed for mouse GrB protein (rat anti-mouse GrB, clone 16G6, eBioscience, CA, USA), as previously described [12]. Equal protein loading was confirmed by re-probing the blot with an anti-mouse β-actin antibody (Sigma-Aldrich, USA).

### Viral infections

#### Murine cytomegalovirus

For pathogenesis studies, mice were inoculated intraperitoneally (ip) with 2×10^4^ plaque-forming units (pfu) of salivary gland propagated (SGV) MCMV-Δm157 mutant virus [48]. SGV stocks were diluted in phosphate-buffered saline-0.05% fetal bovine serum. A dose of 5×10^3^ pfu was used for phenotypic analysis of lymphocytes.

#### Ectromelia virus

Mice were inoculated with the Moscow strain of ECTV subcutaneously (sc) with 10^5^ pfu of virus in the flank of the left hind limb (hock) of mice under avertin anesthesia. All animals were monitored daily for clinical signs of disease, weighed every 2–3 days and euthanized if they lost 25% of their original body weight and recorded as dead the following day.

### Viral quantification

MCMV viral titers in organs were determined by plaque assay using M210B4 cells as previously described [49].

ETCV genome copies in blood were measured by quantitative real time PCR (qRT-PCR) to amplify the target sequence of ECTV-Mos-156 gene, as described elsewhere [50]. Oligonucleotide primers used were, forward: CGCTACACCTTATCCTCAGACAC, and reverse: GGAATTGGGCTCCTTATACCA. Viral DNA was prepared using QiaAmp DNA Mini Kit (Qiagen Pty Ltd, Victoria, Australia) as per manufacturer's instructions. Serial dilutions of a plasmid encoding ECTV-Mos-156 were used as the standard. The qRT-PCR reaction was carried out in SYBR iQ Supermix (Bio-Rad Laboratories), in a total volume of 20 µl using the iQ5 cycler (Bio-Rad Laboratories Pty Ltd, New South Wales, Australia).

### Flow cytometry

Single-cell suspensions were prepared by perfusing the liver via the portal vein with phosphate buffered saline (PBS). The liver was then digested with collagenase buffer (RPMI/2% FCS, 1% (w/v) collagenase IV (GIBCO) for 20 min before being passing through steel mesh. The resulting preparation was resuspended in in a 37.5% isotonic Percoll solution (Pharmacia) and centrifuged at 690 g for 12 min to separate lymphocytes from hepatocytes. Red blood cells were osmotically lysed using NH_4_Cl and cells washed in FACS buffer. Antibodies used Antibodies used for analysis (TCRβ, CD8, CD4, CD11b, CD11c, Ly6C, Ly6G) were purchased from BD Biosciences and the M45 tetramer was obtained from ImmunoID Tetramers, University of Melbourne, Australia. Dead cells were excluded from analysis using propidium iodide.

### Cytokine quantification

IFNγ and TNFα levels in the liver were measured by standard sandwich enzyme-linked immunosorbent assay (ELISA) with antibodies from BD Biosciences. Detection was achieved with poly-horseradish peroxidase (poly-HRP) conjugated to streptavidin (CBL, Amsterdam, Netherlands) and K-Blue (Elisa Systems, Brisbane, Australia).

### Histological analysis

Organs were removed from mice at the designated times and fixed in 10% buffered formal saline. Organs were then embedded in paraffin, tissue sections prepared and sections stained with haematoxylin and counter stained with eosin. IE1 protein was detected by staining slides with an anti-IE1 monoclonal antibody (Clone Chroma 101), and detected with goat anti mouse HRPO and metal enhanced DBA substrate (Thermo Fisher).

### Cell death assays

Cell death induced by perforin (0.135–1.3 nM) and the recombinant mouse inbred and the outbred wild GzmB (12.5–25 nM) was assessed by ^51^Cr release assays as previously described [51].

Cytotoxic activity of M45 specific T cells was assessed by preparing a single-cell suspension from the spleens. Splenocytes were diluted 2-fold on 96-well plates starting with 1×10^6^ cells/well and ^51^Cr-labeled EL4 cells pulsed with M45 peptide (1×10^4^ cells/well) were added. Each assay was performed in triplicate. Chromium release was measure after 4 h incubation. Data are presented as percentage of specific lysis, calculated by the following formula: percentage specific lysis = (experimental c.p.m.−spontaneous release c.p.m.)/(total c.p.m.−spontaneous release c.p.m.)×100.

The capacity of activated CD8 T cells to kill MCMV infected cells was assessed using IC-21 macrophages. B6 or GzmB^W/W^ mice were infected with Δm157 virus and spleens removed at day 6 post-infection. CD8 T cells were purified from the spleen using a CD8a positive selection kit (Stem Cell Technologies) according to the manufacturers instructions. IC-21 macrophages were infected with MCMV 12 h prior to co-culture with the purified CD8 T cells. Cell viability was quantified after 16 h of co-culture by MTT assay.

### Statistical analysis

*In vitro* assays were assessed using a one way ANOVA with Tukey's multiple comparison test. Statistical analysis of survival curves were performed using the Log Rank test. Differences in viral replication within organs were assessed using a two-tailed Mann-Whitney test. Statistical tests were performed using the statistical software package InStat (GraphPad Software, San Diego California USA).

## Supporting Information

Figure S1 **Comparison of human GzmB, GzmB^W^ and GzmB^P^ subsite specificity by anchored substrate phage display.** Cleavage of substrates occurs between the anchored P1 Asp and P1′ residues (arrow). Y-axis shows Δσ values as a percentage, normalised to the highest score (except for the fixed residue for each data set). Numbers of phage sequenced: hGzmB 102; GzmB^W^ 71; GzmB^P^ 70. The sequencing results were analyzed to determine the statistical distribution of each amino acid at each position. In a binomial distribution of amino acids, Δσ yields the difference of the observed frequency from the expected frequency in terms of standard deviations (for methods see [10], [43]).
(TIF)

Figure S2 **ETCV infection in GzmB^w/w^ mice.** (**A**) B6 mice (black square) or GzmB^w/w^ mice (white square) were infected with 1×10^5^ pfu of ECTV and survival monitored. n≥5 (**B**) Blood was isolated from mice at day 8 post-infection, and ECTV quantified by qRT-PCR. Mean ± SEM are plotted, where n≥5.
(EPS)

Figure S3 **MCMV infection in Serpinb9^−/−^ mice.** B6 mice or Serpinb9^−/−^ mice were infected with 2×10^4^ pfu of MCMV Δm157. At day 6 post-infection, viral loads in the spleen, liver, and lungs were quantified by plaque assay. Data are pooled from 2 independent experiments, mean ± SEM are plotted, where n≥6.
(EPS)
