# Supplementary figures and images for: A Natural Genetic Variant of Granzyme B Confers Lethality to a Common Viral Infection
Source: PLoS Pathog. 2014 Dec 11;10(12):e1004526. doi: 10.1371/journal.ppat.1004526 (PMC4263754; doi:10.1371/journal.ppat.1004526)

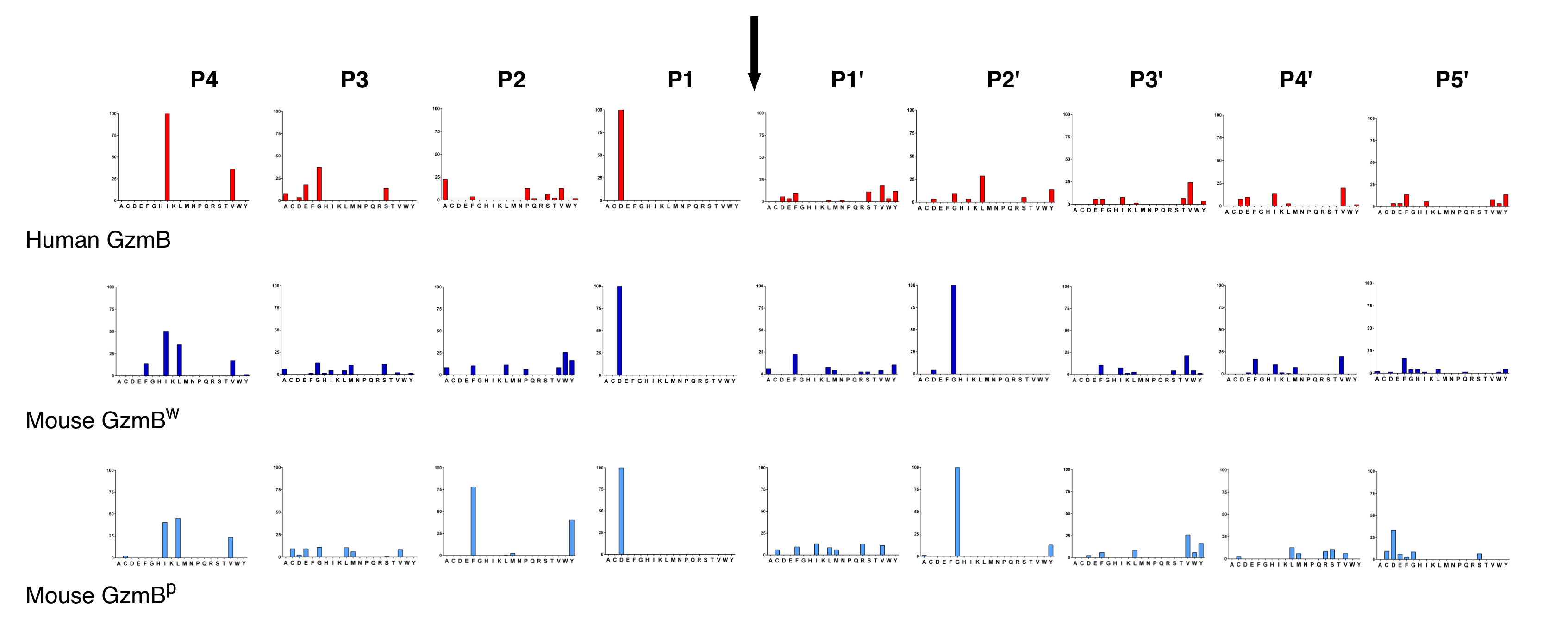

Supplement: Figure S1 — Comparison of human GzmB, GzmBW and GzmBP subsite specificity by anchored substrate phage display. Cleavage of substrates occurs between the anchored P1 Asp and P1′ residues (arrow). Y-axis shows Δσ values as a percentage, normalised to the highest score (except for the fixed residue for each data set). Numbers of phage sequenced: hGzmB 102; GzmBW 71; GzmBP 70. The sequencing results were analyzed to determine the statistical distribution of each amino acid at each position. In a binomial distribution of amino acids, Δσ yields the difference of the observed frequency from the expected frequency in terms of standard deviations (for methods see [10], [43]). (TIF) [file ppat.1004526.s001.tif]
